# Unveiling the Molecular Mechanisms of Bisphenol-Induced Olfactory Toxicity in Zebrafish

**DOI:** 10.3390/toxics14090776

**Published:** 2026-08-31

**Authors:** Yaxuan Li, Jing Cao, Haoliang Jiang, Mei Hou, Xuefeng Hu, Liting Chen, Dan Li, Yongju Luo

**Affiliations:** 1 The Research Institution of Beautiful China and Ecological Civilization (A University Think Tank of Shanghai Municipality), Shanghai Institute of Technology, Shanghai 201418, China; liyaxuan127@163.com (Y.L.); 15629343669@163.com (J.C.); jhl2622744588@163.com (H.J.); hm13275326626@163.com (M.H.); xuefenghu@163.com (X.H.); 2 Guangxi Academy of Fishery Sciences, Nanning 530021, China; 3 Faculty of Urban Construction and Ecological Technology, Shanghai Institute of Technology, Shanghai 201418, China; chenlt3433@126.com (L.C.); lfylzc123@163.com (Y.L.)

**Keywords:** histopathology, transcriptomics, molecular dynamics, olfactory, bisphenol analogues

## Abstract

Bisphenol analogues, including bisphenol F (BPF) and bisphenol S (BPS), are increasingly used as alternatives to bisphenol A (BPA), yet their potential olfactory effects remain poorly understood. In this study, adult zebrafish (*Danio rerio*) were exposed to environmentally relevant concentrations (5 μg/L) of BPF and BPS for 28 days to investigate their impacts on the olfactory system. Histopathological assessment revealed changes in olfactory epithelial morphology, particularly in the BPF-exposed group. Transcriptomic analysis identified 3260 and 1602 differentially expressed genes in BPF and BPS exposure groups, respectively, involving pathways related to signal transduction, immune response, endocrine regulation, cell apoptosis, and olfactory signaling. Further molecular docking and 200 ns molecular dynamics simulations demonstrated stable interactions between with BPF exhibiting stronger binding affinities and more hydrophobic contacts with the targeted proteins (OR, GNB2 and p53) among the targeted proteins. Binding free energy calculations confirmed BPF’s greater binding potential. These findings suggest that BPF may exert more potent olfactory toxicity than BPS, disrupting signal transmission and sensory function in aquatic species. This study provides new mechanistic insights into the molecular basis of bisphenol-induced olfactory disruption and emphasizes the ecological risks of BPF and BPS in aquatic environments.

## 1. Introduction

Bisphenol analogs serve prominent roles as modifiers, stabilizers, and photoinitiators in the production of epoxy resins and polycarbonate plastics [1]. The annual output of BPA is approximately 10 million tons [2], whereas its main analogs, BPS and BPF, are produced in quantities of around 100,000 tons and 10,000 tons, respectively [3,4]. As numerous jurisdictions implement regulations to phase out BPA, the utilization of BPF and BPS has seen a marked increase. These compounds are prevalent in aquatic ecosystems, resulting from their release during production processes, incomplete removal in wastewater treatment, leaching from landfills, and degradation of materials containing BPF and BPS [2]. In the Turag River, Bangladesh, BPF concentrations have been reported to range from 1.12 to 7.31 µg/L [5], whereas the maximum detected concentration of BPS in the Adyar River, India, was 7.20 µg/L [6]. The half-life of BPF in surface waters is reported to be approximately 44.0 days, with BPS demonstrating significant persistence and minimal degradation under natural aquatic conditions [7]. Due to their environmental stability and widespread distribution, bisphenols have the potential for bioaccumulation in aquatic life. Indeed, BPF and BPS were detected in 26.2% and 50.8% of wild fish samples collected from the Yangtze River Basin, respectively [8]. Additionally, BPF and BPS have been detected in various aquatic organisms across different regions. For instance, mean concentrations of 30.9 and 50.6 ng/g, respectively, were reported in 20 species collected from the Pearl River Estuary [9]. Similarly, in clam samples collected from the Sicilian Lagoon, the average concentrations of BPF and BPS were 3.3 and 7.1 μg/kg, respectively [10].

BPF and BPS have been identified as neuroendocrine-disrupting chemicals, exhibiting reproductive and neurotoxic effects. They can increase the number of gonadotropin-releasing hormone 3 neurons via estrogen receptor and aromatase-mediated pathways, upregulate reproductive neuroendocrine-related genes (e.g., *lhβ*, *fshβ*, *erα*, *erβ*, *cyp19a*), and elevate hormone levels, including LH, FSH, E2, and GH [11]. Additionally, BPF has been shown to activate microglia, induce inflammation, and cause neuronal cell loss in zebrafish [12]. The olfactory system is a crucial component of the nervous system and plays a fundamental role in sensory processing [13,14]. It enables fish to detect and recognize trace substances in the water across vast areas and long distances, might triggering stress-related behaviors in response to environmental stimuli [14]. Olfactory information is relayed from olfactory receptors to the olfactory bulb, and subsequently through olfactory nerves to the olfactory cortex for processing. This pathway is vital for various fish behaviors, including foraging, reproduction, and predator avoidance [14]. Exposure to environmental contaminants has been shown to impair the olfactory system. For example, dithiocarbamates reduce the number of olfactory sensory neurons and the activity of cAMP in fish olfactory tissues. These changes regulate G-protein (gpαolf) and CaCC-related genes (e.g., *pvalb8*, *camk2d*), leading to an increase in cup cells and mucus production, which diminishes olfactory sensitivity and impairs predator-avoidance responses [13,14,15,16,17,18]. Similarly, methyl parathion decreases acetylcholinesterase levels, inhibits glutamatergic neurotransmission in the olfactory system, and downregulates genes involved in neuronal function and signal transduction, thus disrupting olfactory behavior [16,17,18]. Moreover, ethynylestradiol has been found to activate estrogen-responsive cells in the olfactory bulb, promoting the formation of olfactory glomeruli and inhibiting olfactory-mediated neuronal activity by suppressing intrinsic excitability, thereby impairing the ability to recognize stimulants [19]. However, there remains limited research on the neuroendocrine-disruptors (such as bisphenols) on the fish olfactory.

Molecular docking and molecular dynamics simulations represent advanced computational methodologies increasingly utilized in ecotoxicological research, particularly for elucidating the interactions between environmental contaminants and biological mechanisms. Molecular docking facilitates the prediction of binding affinities between contaminants and specific biological targets, including enzymes, receptors, and proteins, thus aiding in the identification of potential toxicity pathways [20]. Conversely, molecular dynamics simulations offer a comprehensive, time-dependent perspective of molecular interactions by modeling the trajectories and behaviors of atoms and molecules over time [21]. These simulations are instrumental in investigating the impact of contaminants on protein conformation, receptor activity, and enzyme functionality. Specifically, by simulating the interaction between bisphenols and designated receptors involved in fish olfaction, molecular dynamics simulations can substantiate and elucidate the molecular mechanisms.

To bridge this knowledge gap, the present study explores the olfactory toxicity of BPF and BPS in zebrafish through a multidisciplinary framework that encompasses histopathological assessment, transcriptomic analysis, molecular docking, and molecular dynamics simulations. The outcomes are anticipated to yield pivotal insights into the molecular pathways responsible for olfactory impairments induced by neuroendocrine-disrupting chemicals, thereby underscoring their potential hazards to aquatic ecosystems.

## 2. Materials and Methods

### 2.1. Chemicals and Treatments

BPF (CAS 620-92-8, ≥98%) and BPS (CAS 80-09-1, ≥98%) were obtained from Sigma-Aldrich (St. Louis, MO, USA). All solvents utilized in this study were of HPLC grade. Stock solutions of BPF and BPS (10 g/L) were prepared by dissolving each compound in dimethyl sulfoxide (DMSO, 100%; Sigma-Aldrich) and stored at 4 °C. Based on reported environmental concentrations of BPF and BPS in aquatic systems (up to ~7 μg/L), three treatment groups were established: Control, 5 μg/L BPF, and 5 μg/L BPS, each with 3 replicates. Exposure solutions were prepared by serial dilution of the stock solutions with the test medium. Stock solutions of BPF and BPS were prepared in DMSO at 1 mg/mL. For exposure, 15 μL of each stock solution was added to 3 L of test medium to obtain a final concentration of 5 μg/L. The same volume of DMSO was added to the control group. The actual concentrations of the exposure solutions were measured at 0 and 24 h after the initial exposure, as confirmed by LC-MS/MS analysis.

### 2.2. Animals and Exposure

Adult zebrafish were fed live brine shrimp (*Artemia nauplii*) twice daily and maintained in a flow-through aquarium system following standard national guidelines, with all procedures approved by the Institutional Animal Care and Use Committee of Shanghai Institute of Technology (SIT-2025-LL44). The rearing conditions were as follows: pH 8.5, water hardness 6–8, temperature 28 ± 1 °C, dissolved oxygen 6 mg/L, and a 14 h light:10 h dark photoperiod. Adult fish (4 months old) were kept at a male-to-female ratio of 2:1 and transferred to mating tanks for spawning. Fertilized eggs were collected and rinsed with embryo medium [22], and raised until day 150 in the flow-through aquarium system. A total of 200 zebrafish were selected from these adults, acclimated for one week, after which they were rinsed with 12 L of filtered, dechlorinated tap water and randomly assigned to nine 15 L tanks (20 fish per tank) for exposure, and the remaining 20 fish were held in reserve to account for potential mortalities. Water was renewed every 24 h. After 28 days of exposure, the fish were anesthetized using MS-222 (150 mg/L). Olfactory tissues were then dissected, with portions fixed in 4% paraformaldehyde for histopathological analysis and the remainder stored at −80 °C for transcriptome sequencing.

### 2.3. Chemical Analysis

Water samples were centrifuged at 3500 rpm for 5 min and subsequently filtered through 0.22 μm membranes. BPF and BPS were separated on an ACQUITY UPLC™ BEH C18 column (2.1 mm × 150 mm, 1.7 μm) using an ACQUITY Ultra Performance LC system (Waters Corp., Milford, MA, USA) coupled to a Xevo TQ-S triple quadrupole mass spectrometer(Waters Corp., Milford, MA, USA) equipped with an electrospray ionization (ESI) source operating in positive mode. The mobile phase for gradient elution consisted of Milli-Q water (A) and methanol (B). The gradient program was as follows: initial 35% B at 0 min, increased to 70% B at 2 min, then stepped to 95% B and maintained for 1 min at a flow rate of 0.50 mL/min. The column temperature was maintained at 40 °C, and the injection volume was 5 μL.

### 2.4. Histology

One zebrafish olfactory tissues were collected from each tank and fixed in 4% paraformaldehyde for 12 h at room temperature at the end of the 28-day exposure period (three tissue for each group). The fixed tissue was washed repeatedly in 70% ethanol, followed by dehydration through a graded series of ethanol solutions, and cleared with xylene. The tissue was then immersed in paraffin (56–58 °C, Merck, Darmstadt, Germany) and subjected to a constant-temperature vacuum paraffin embedding bath for 1 h, with a 45-min immersion period. Continuous 4 µm thin sections of the paraffin blocks were obtained using a rotary microtome. The sections were deparaffinized with xylene, rehydrated through a graded ethanol series, and further hydrated with distilled water. Hematoxylin staining was followed by counterstaining with 1% eosin. The stained slides were dehydrated through graded ethanol, cleared with xylene, and mounted with DPX. Finally, the slides were photographed using a digital slide scanner (3D Histech, Budapest, Hungary)).

### 2.5. Transcriptomic Analysis

Total RNA was extracted and purified from 3 olfactory tissues in each replicate using an RNA extraction kit (Vazyme Biotech Co., Ltd., Nanjing, China), adhering to the manufacturer’s guidelines. The quality of the RNA was evaluated with a NanoDrop™ (Thermo Fisher Scientific, Waltham, MA, USA) and Agilent 2100 (Agilent Technologies, Santa Clara, CA, USA). Only high-quality RNA samples (28S:18S ratio = 2.0–2.2, RIN > 9.0) were used for library construction with a commercial kit (Mega Genomics, Beijing, China). The libraries were then sequenced on the Illumina NovaSeq 6000 platform (Illumina, Inc., San Diego, CA, USA) using 150-cycle paired-end sequencing (300 total cycles). The resulting read counts were normalized to FPKM (Fragments Per Kilobase of transcript per Million mapped reads) values. Differentially expressed genes (DEGs) between groups were analyzed using DESeq2 software (version 1.42.0), and fold changes (log_2_) were calculated by comparing the FPKM values of the treatment and control groups. Gene Ontology (GO) and Kyoto Encyclopedia of Genes and Genomes (KEGG) pathway enrichment analyses were performed using GOATOOLS [23] and KOBAS [24], respectively, to annotate gene functions and identify biological pathways associated with DEGs. The sample quality control in transcriptomics analysis was detailed in Text S1 (Supplementary Materials).

### 2.6. Binding Affinity and Interaction Modes

To explore the potential molecular interactions through which BPF and BPS may affect the zebrafish olfactory system, molecular docking were conducted using AutoDock Vina (Scripps Research, La Jolla, CA, USA) against selected proteins, which were selected from key genes identified in deferentially expressed pathways in transcriptomic analysis conducted in this study. Zebrafish (*Danio rerio*) protein structures were retrieved from the UniProt database (https://www.uniprot.org/), and 3D structures of BPF and BPS were obtained from the PubChem database (https://pubchem.ncbi.nlm.nih.gov/). Since experimentally resolved structures for most selected zebrafish proteins were unavailable in the Protein Data Bank (PDB), AlphaFold-predicted structures from the AlphaFold Protein Structure Database were used. The structural confidence of the predicted models was evaluated before docking analysis (Table S2). For each protein–ligand pair, the binding affinity was calculated, and the lowest-energy binding pose was selected for further analysis by molecular dynamics.

### 2.7. Molecular Dynamics Simulation

To assess the stability of the interactions between BPF and BPS and the target proteins in a realistic environment over time, molecular dynamics (MD) simulations were conducted using GROMACS 2023.2 for 200 ns. The topologies of small molecules were generated using the Sobtop tool in combination with the GAFF force field (Tian. 2024), while protein topologies were prepared using the AMBER ff14SB force field via the pdb2gmx module. Each protein-ligand complex was solvated in a TIP3P cubic water box with a 1.0 nm buffer distance, and Na^+^/Cl^−^ ions were added to neutralize the system. Energy minimization was carried out using the steepest descent and conjugate gradient algorithms. Electrostatic interactions were treated with the PME method, with 1.0 nm cutoffs for both electrostatic and van der Waals interactions. The system was equilibrated for 200 ps under NVT (300 K, V-rescale) and subsequently under NPT (1 atm, Berendsen) conditions, followed by a 200 ns production run under periodic boundary conditions. Simulation trajectories were analyzed to evaluate the stability, conformational dynamics, and interaction mechanisms of the protein-ligand complexes. Binding free energies were quantified using the molecular mechanics/Poisson-Boltzmann surface area (MM-PBSA) method. Binding modes and intermolecular interactions, such as hydrogen bonding, were visualized and analyzed using PyMOL(Schrödinger, LLC, New York, NY, USA) and the PLIP tool(PharmAI GmbH, Dresden, Germany) [25].

### 2.8. Statistical Analyses

For morphometric analysis, epithelial thickness and lamellar width were measured from HE-stained sections using ImageJ software(version 1.54u8, U.S. National Institutes of Health, Bethesda, MD, USA). Three biological replicates per group were included. Multiple olfactory lamellae were measured for each replicate, and their average was used as a single independent data point. Measurements were performed in a blinded manner by two independent observers, and the mean of the two observers’ measurements was used for analysis.

Data are presented as mean ± SEM. Statistical analysis was performed using GraphPad Prism software(version 11.1.0, GraphPad Software, LLC, Boston, MA, USA). Differences among groups were analyzed by one-way analysis of variance (ANOVA), followed by the post-hoc least·significant difference (LSD) test to compare each exposure group with the control group. A value of *p* < 0.05 was considered statistically significant.

## 3. Results and Discussions

The measured concentrations of BPF and BPS in the control group were below the detection limit. In the exposure groups, BPF levels were 5.34 ± 0.30 μg/L at 0 h and 4.90 ± 0.09 μg/L at 24 h, while BPS levels were 5.34 ± 0.31 μg/L at 0 h and 5.12 ± 0.37 μg/L at 24 h. These concentrations were consistent with the nominal exposure levels of 5.00 μg/L for both BPF and BPS (Table 1).

### 3.1. Effects of BPF and BPS on the Olfactory Histopathology in Zebrafish

**Figure 1:**
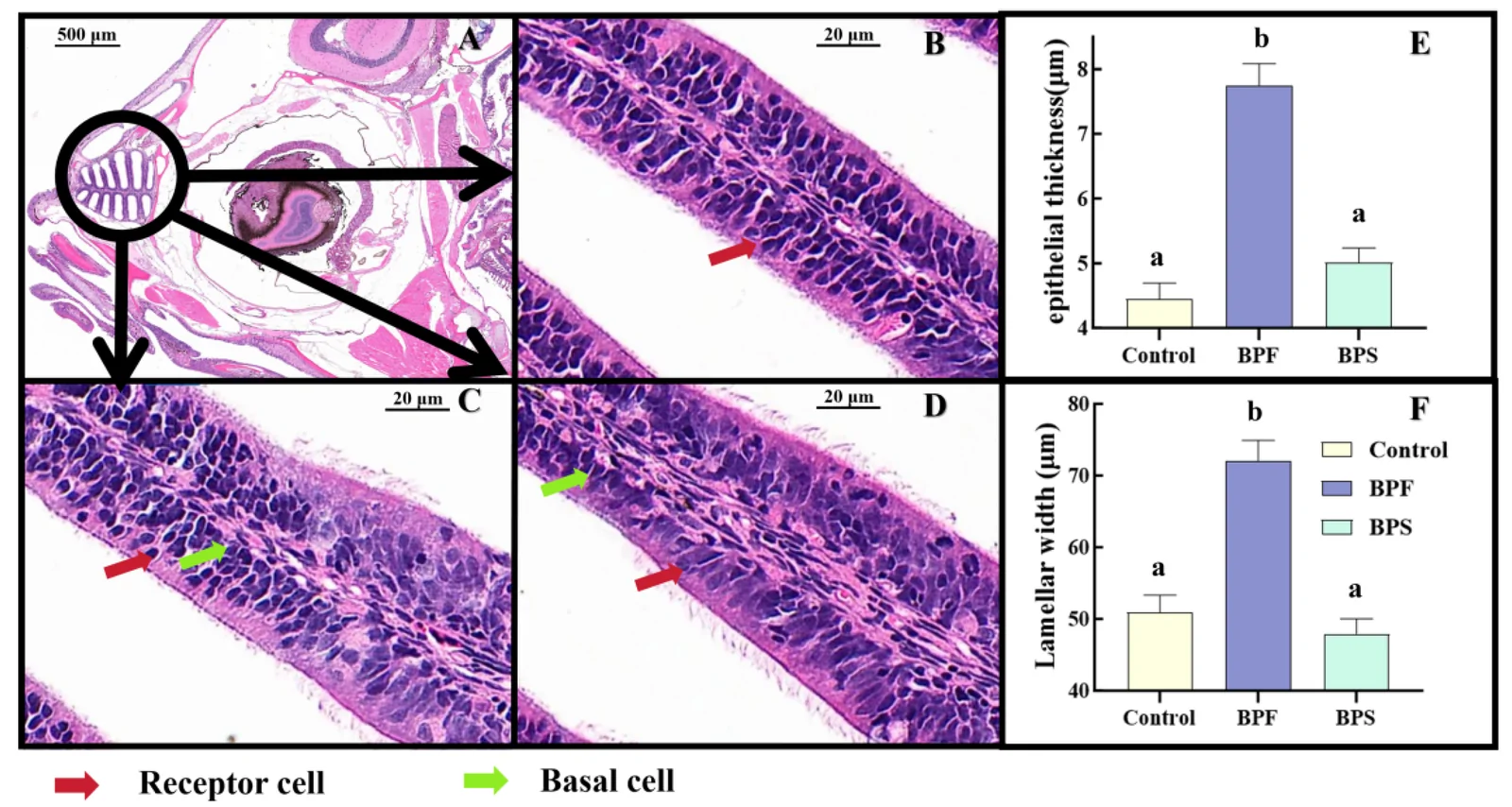
Representative H&E-stained images and morphometric analysis of the olfactory tissue of zebrafish (*Danio rerio*) after BPF and BPS exposure. (**A**) Low-magnification image showing the olfactory rosette. (**B**–**D**) Representative high-magnification images of olfactory lamellae from the control, BPF, and BPS groups, respectively. Red arrows indicate receptor cells, and green arrows indicate basal cells. (**E**) Quantification of olfactory epithelial thickness. (**F**) Quantification of lamellar width. Data are presented as mean ± SEM (*n* = 3). Different letters indicate significant differences among groups (*p* < 0.05).

Histopathological analysis and morphometric quantification revealed slightly alterations in the OE, particularly in the BPF-exposed group, after 28 days of exposure. In the control group, the OE maintained a normal structure, with cells in each layer arranged neatly and no apparent lesions (Figure 1A,B; Figure S1). In the BPF- and BPS-exposed groups, slight changes were observed in the olfactory lamellae, including minor increase epithelial thickening and lamellar widening (Figure 1C,D; Figure S1). Quantitative analysis further showed that the epithelial thickness increased from 4.4 μm in the control group to 7.9 μm in the BPF group, while the lamellar width increased from 50.9 μm to 72.1 μm (Figure 1E,F). In the BPS group, changes were minimal, with an epithelial thickness of 4.9 μm and a lamellar width of 47.9 μm, and no significant difference was detected compared with the control group (Figure 1E,F). These results indicate that BPF exposure induced more alterations in the zebrafish olfactory epithelium than BPS exposure under the present experimental conditions, where receptor cell swelling was observed.

The histopathological changes observed in the zebrafish olfactory epithelium following exposure to BPF and BPS may be associated with pathways related to signal transduction, immune system responses, and apoptosis [13,15,26,27]. The increase in epithelial thickness and lamellar width induced by BPF/BPS, as a defensive strategy, elevates mucus production to provide additional protection against the harmful effects of contaminants on epithelial cells [28]. However, excessive mucus can cover olfactory receptors, reducing olfactory sensitivity and consequently affecting the transmission and processing of olfactory signals. The irregular distribution of receptor cells, particularly in the BPF-exposed group, could reflect a disruption of normal regenerative processes [15]. BPF and BPS, as known neuroendocrine sisruptors [12,13,14,19], may interfere with hormonal signaling pathways, affecting cellular differentiation and regeneration in the OE. These alterations may impair olfactory signal transmission, leading to a reduced sensitivity to changes in the external environment. The specific mechanisms underlying these effects warrant further investigation.

### 3.2. Effects of Transcriptome Profiles in Zebrafish Olfactory

**Figure 2:**
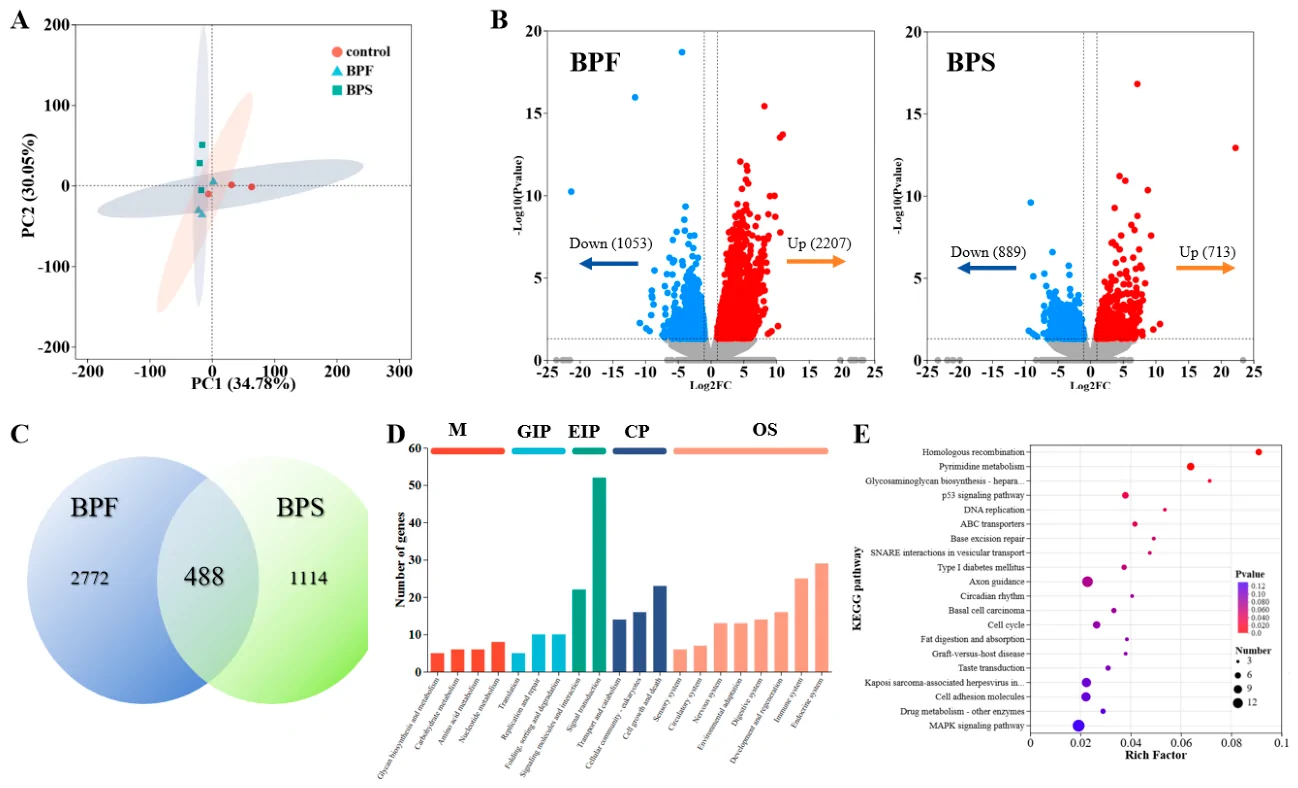
Transcriptomic Profiling of Zebrafish Olfactory Tissues Following 28-Day Exposure to BPF and BPS (*n* = 3). (**A**) PCA plot showing sample distribution among Control, BPF, and BPS groups. (**B**) Volcano plots of differentially expressed genes (DEGs) in BPF (**left**) and BPS (**right**) groups vs. Control. Red/blue dots indicate up/down-regulated genes; numbers show significant DEGs. (**C**) Venn diagram of shared and unique DEGs between BPF and BPS groups. (**D**) Functional classification of DEGs by Gene Ontology (GO) terms across biological processes. (**E**) KEGG pathway enrichment bubble plot. Dot size/color represent gene count and p-value, respectively.

The transcriptomic analysis showed that the quality-trimmed reads for all samples ranged from 41.41 to 45.42 million, with Q20 values exceeding 97.59% (Table S1). The PCA analysis reveals differences in the overall transcriptomic profiles of olfactory tissues among the control, BPF, and BPS treatment groups. PC1 and PC2 explained 34.78% and 30.05% of the total variance, respectively, accounting for 64.83% of the total variance combined (Figure 2A). Differential gene expression analysis identified 3260 differentially expressed genes (DEGs) between the BPF and control groups, comprising 2207 upregulated and 1053 downregulated genes, whereas 1602 DEGs were detected between the BPS and control groups, including 713 upregulated and 889 downregulated genes (Figure 2B). Venn analysis further showed that 488 DEGs were shared between the BPF and BPS treatments, while 2772 and 1114 DEGs were specific to the BPF and BPS groups, respectively (Figure 2C). Thus, the total numbers of DEGs correspond to 2772 BPF-specific plus 488 shared DEGs (3260 in total) and 1114 BPS-specific plus 488 shared DEGs (1602 in total). The annotated pathways revealed that these 488 DEGs are primarily associated with signal transduction, endocrine, immune system regulation, cell growth and death, and signaling molecule interactions (Figure 2D). KEGG enrichment analysis further identified TOP5 significantly affected pathways, including homologous recombination, pyrimidine metabolism, glycosaminoglycan biosynthesis (heparan sulfate/heparin), the p53 signaling pathway, and DNA replication (Figure 2E).

Previous studies have demonstrated that BPF and BPS interfere with estrogen signaling and signal transduction pathways, thereby affecting reproduction, and the nervous system in the gonads, intestine, and brain tissues of fish [28]. Our transcriptomic results extend these observations to the olfactory tissue of zebrafish, showing that BPF and BPS exposure affected pathways related to signal transduction, endocrine regulation, immune function, cell growth and death, and signaling molecule interactions. These altered pathways are not independent events but may be mechanistically connected. Because the olfactory epithelium is a sensory neural tissue, disruption of endocrine-related signaling and intracellular signal transduction may influence odorant recognition, neuronal communication, epithelial homeostasis, and cellular stress responses. Similarly, triclosan and triclocarban impair the olfactory capacity of goldfish by hindering odorant recognition, disrupting olfactory signal transduction, and interfering with olfactory information processing [29]. Tetrabromobisphenol A, at environmentally relevant levels, negatively affects the olfactory sensitivity of aquatic organisms by interfering with the transmission of aqueous stimuli to olfactory receptors, impeding the binding of odorants to their receptors, and disrupting the olfactory signal transduction pathway [27]. Although BPF and BPS are structurally distinct from TBBPA, these studies provide a mechanistic framework suggesting that bisphenol-related compounds may impair olfactory function through convergent pathways involving receptor-mediated odorant detection, G protein-associated signal transduction, endocrine disruption, and cellular stress responses. Therefore, the present transcriptomic findings suggest that BPF and BPS may affect zebrafish olfactory function not only through general endocrine-disrupting activity but also through direct interference with olfactory signaling and epithelial cellular responses.

### 3.3. Molecular Docking

**Figure 3:**
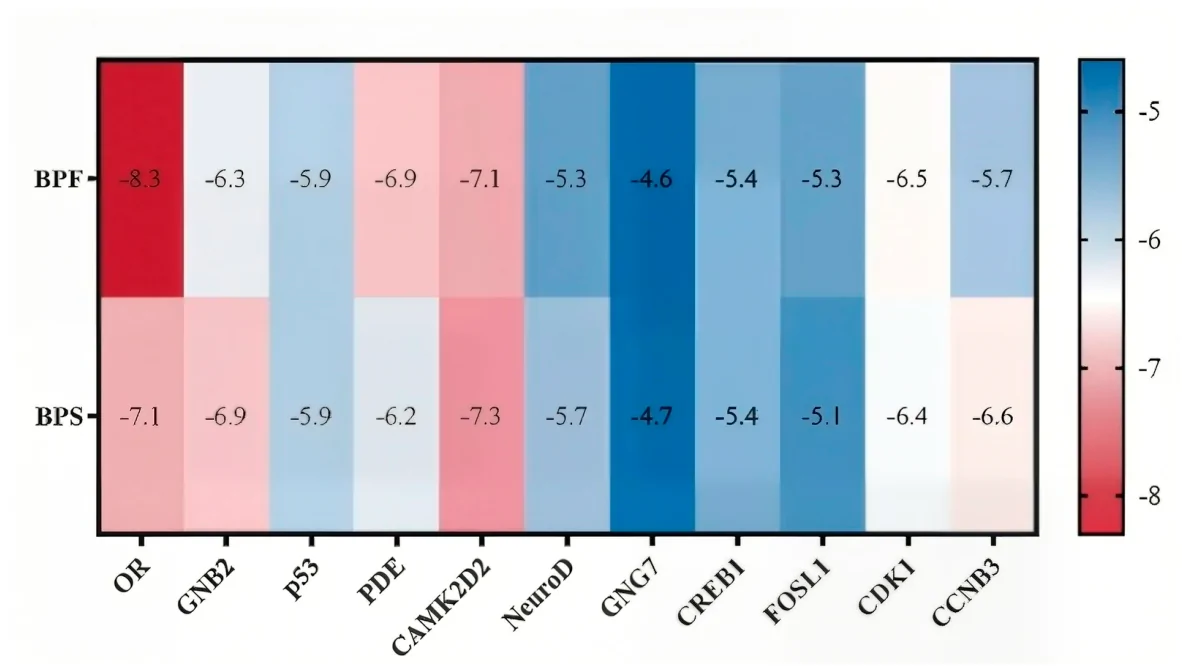
Heatmap of predicted binding affinities between BPF/BPS and candidate target proteins using AutoDock Vina.

Based on the KEGG functional annotation and enrichment analysis of transcriptomic data, 11 target proteins were selected from the target pathways for molecular docking analysis. The target proteins were selected based on key genes identified from significantly enriched pathways in the transcriptomic analysis conducted in this study. These included 6 proteins involved in the signal-related pathway: PDE (Phosphodiesterase), CAMK2D2 (Calcium/calmodulin-dependent protein kinase II delta 2), NeuroD (Neurogenic differentiation factor), GNB2 (G protein subunit beta 2), GNG7 (Guanine nucleotide-binding protein subunit gamma 7), and OR (Olfactory receptor). Three proteins were associated with the cell growth and death pathway: CDK1 (Cyclin-dependent kinase 1), CCNB3 (G2/mitotic-specific cyclin-B3), and p53 (Cellular tumor antigen p53). In addition, FOSL1 (FOS-like 1, AP-1 transcription factor subunit beta) was involved in the immune system pathway, and CREB1 (Cyclic AMP-responsive element-binding protein 1b) in the endocrine system pathway (detailed descriptions of target proteins listed in Table S2). Although not all selected proteins are directly involved in olfactory signal transduction, these pathways are closely associated with cellular signaling, apoptosis, immune response, and neuroendocrine regulation, which are essential for maintaining the structural and functional integrity of the olfactory system. Protein structures were obtained from the UniProt database, with predicted 3D structures generated by AlphaFold. Molecular docking simulations were performed to investigate the interactions of BPF/BPS and these 11 selected proteins. Detailed results, including binding energy (Eb), types of bond formations, specific bond interactions, and involved amino acid residues, are presented in Figure 3 and Table S3.

Eb as a key stability metric for receptor-ligand complexes, where more negative free energy values correspond to stronger binding affinity and greater complex stability [29,30]. Eb values below −5 kcal/mol generally indicate strong and stable interactions [31]. Eb values between BPF/BPS and the 11 selected target proteins in the zebrafish olfactory system ranged from −5.1 to −8.3 kcal/mol, except for the interaction of BPF (−4.6 kcal/mol) and BPS (−4.7 kcal/mol) (Figure 3 and Table S3). Among these 9 receptors, BPF (45 hydrophobic and 16 hydrogen bonds) bind more bonds with receptor residues than BPS (33 hydrohpobic and 12 hydrogen bonds), indicating that BPF might has a more extensive and stable binding interface than BPS [32].

Olfactory receptor (OR) system in the signal transduction pathway, BPF primarily interacts with nonpolar residues such as ILE, VAL, and TYR, indicating that its binding is largely driven by a broad hydrophobic interface. In contrast, BPS exhibits fewer hydrophobic interactions and instead engages more charged residues (such as GLU and LYS), along with π-cation interactions. These distinct binding profiles may influence the conformation and ultimately the function of the OR protein [33,34,35]. In GNB2, BPF primarily relies on hydrogen bonds with residues ALA, SER, and VAL, whereas BPS mainly forms one hydrophobic interaction with ALA and two hydrogen bonds involving ALA and VAL. Despite relying solely on hydrogen bonds, BPF exhibits slightly stronger binding affinity to GNB2 than BPS. This is likely due to the greater number and optimal positioning of hydrogen bonds, particularly involving the polar residue SER, which enhances binding specificity and stability. In contrast, BPS forms fewer hydrogen bonds and includes a non-directional hydrophobic interaction, resulting in a less stable complex [36].

Molecular docking results revealed that BPF exerts a distinct and potentially stronger influence on zebrafish olfactory-related proteins compared to BPS, which kept consistent with the histopatholoogy and transcriptomic. To further investigate the differences in protein-ligand interactions and their potential impact on signaling pathway modulation, molecular dynamics (MD) simulations were subsequently conducted on the docked complexes.

### 3.4. Molecular Dynamics Simulation

**Figure 4:**
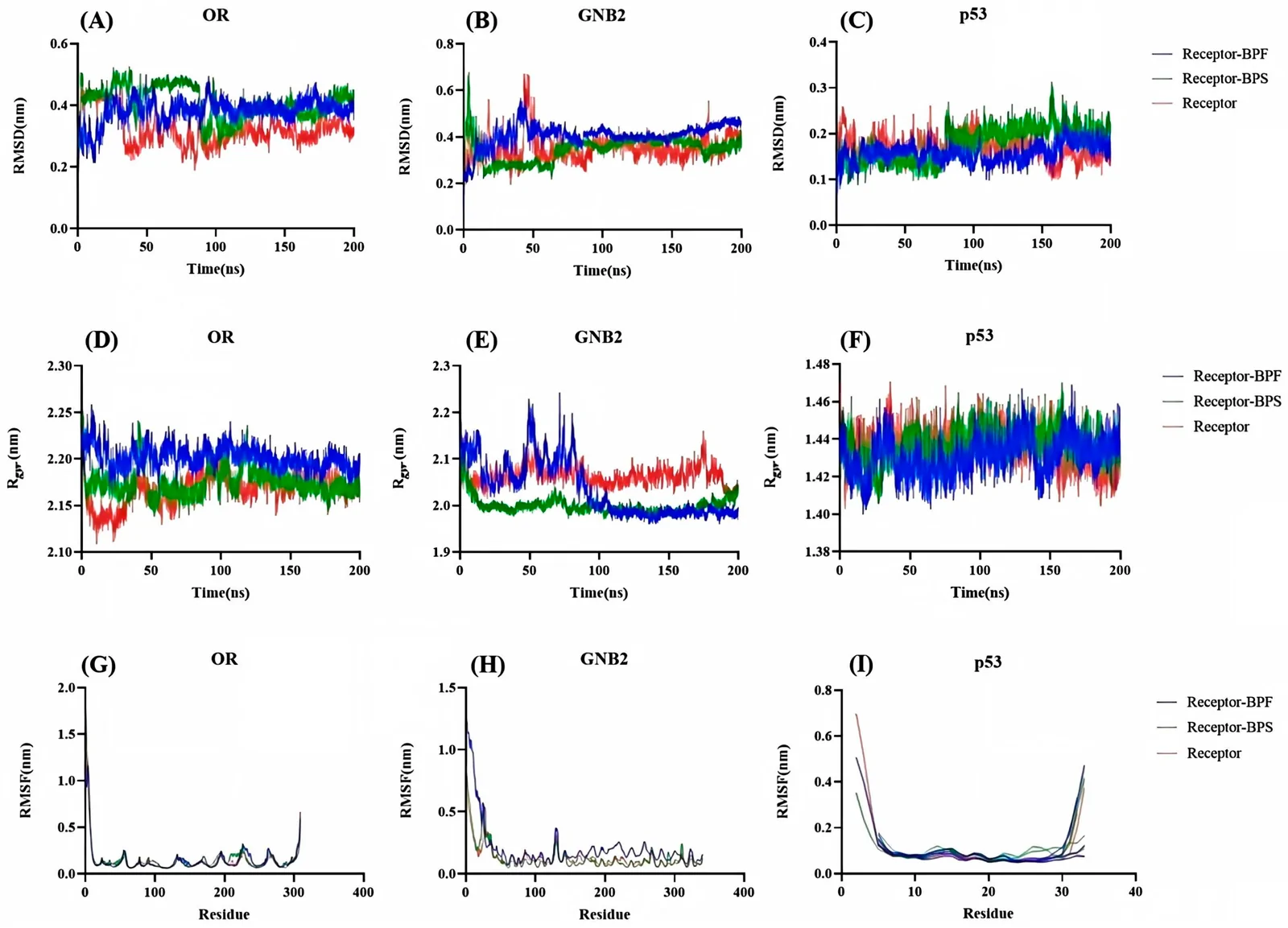
Molecular dynamics analysis of OR, GNB2, and p53 in free states and in complexes with BPF or BPS during 200 ns simulations. Root mean square deviation (RMSD) of the backbone atoms forOR (**A**), GNB2 (**B**), and p53 (**C**) over the 200 ns simulation time. Radius of gyration (RgRg) reflecting the compactness of the protein structures forOR (**D**), GNB2 (**E**), and p53 (**F**). Root mean square fluctuation (RMSF) per residue indicating the flexibility of individual amino acids for OR (**G**), GNB2 (**H**), andp53 (**I**).

MD simulations of 200 ns were performed on the 11 protein–BPF/BPS complexes to evaluate the dynamic behavior and structural stability of the predicted protein–ligand complexes under simulated aqueous conditions (Figure 4 and Figures S2 and S3). Among these systems, the OR, GNB2, and p53 complexes exhibited comparatively stable conformational behaviors and were therefore selected as representative complexes for detailed trajectory visualization and keyframe analysis (Figure 4). The three representative complexes maintained RMSD values below 0.5 nm, reflecting limited deviation of the protein backbone from the initial structure and indicating overall structural stability (Figure 4A–C) [37]. The radius of gyration (Rgyr) measures how protein atoms are distributed around their center of mass and is used to assess structural compactness. Consistent with RMSD results, the OR, GNB2, and p53 systems maintained stable Rgyr values (about 1.5–2.3 nm) in both BPF and BPS systems. (Figure 4D–F). To evaluate residue-specific variations on ligand binding, we computed the root mean square fluctuation (RMSF) metrics for all receptor-ligand complexes. The values of RMSF of OR, GNB2, and p53 systems were below 0.5 nm (Figure 4G–I), indicative of robust structural integrity [38]. Notably, OR and GNB2 exhibit an initial peak (~1.5 nm) at the N-terminus, which decreases across the sequence, while p53 maintains consistently low RMSF values (<0.5 nm) throughout. Together with stable RMSD and Rgyr trajectories, these results suggest that these three representative receptor-BPF/BPS complexes retained overall structural stability during the simulation period [39,40].

**Figure 5:**
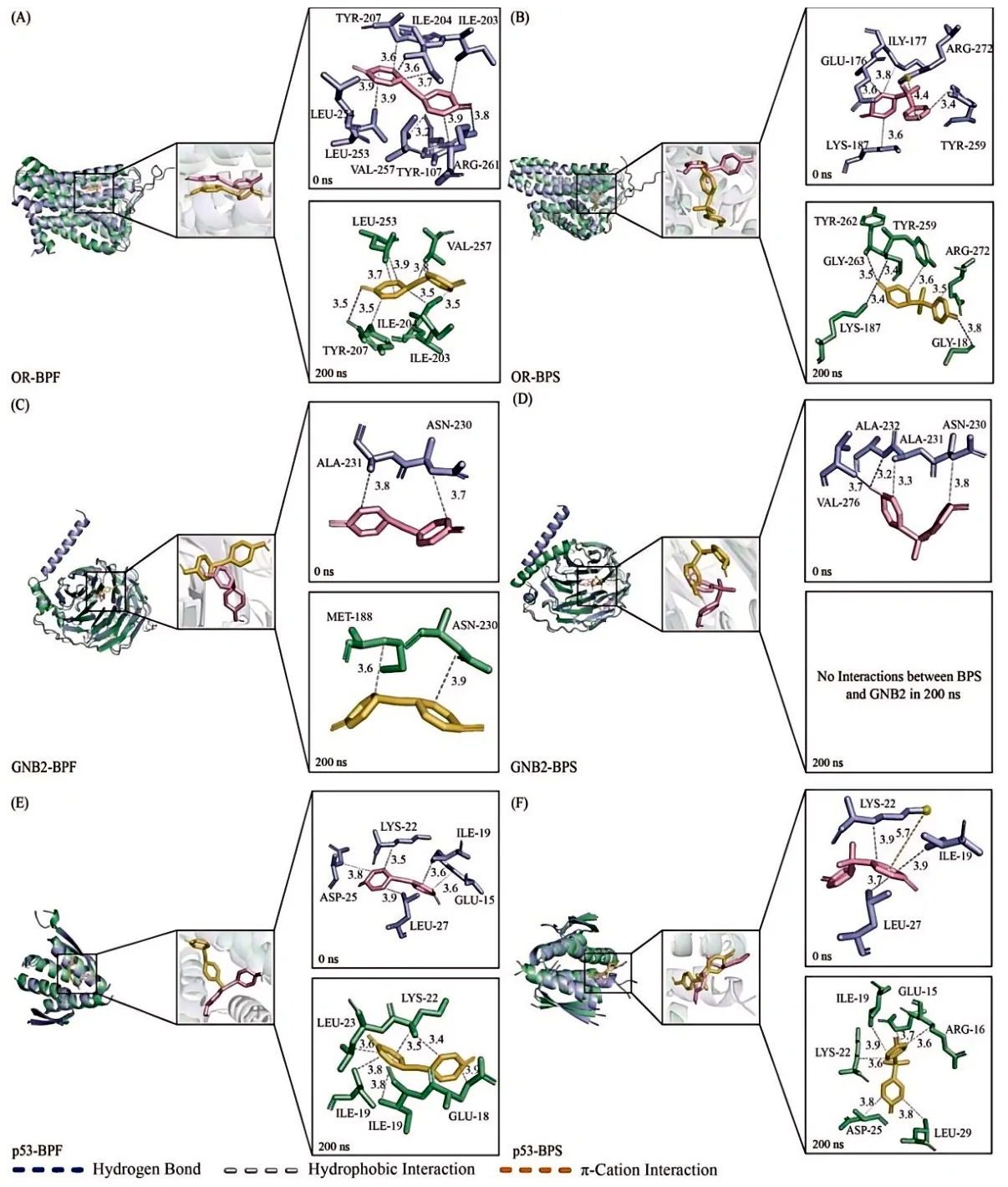
Structural superimposition and interaction analysis of OR, GNB2, and p53 complexes with BPF and BPS before and after 200 ns molecular dynamics simulations. OR-BPF complex (**A**), OR-BPS complex (**B**); GNB2-BPF complex (**C**); GNB2-BPS complex (**D**); p53-BPF complex (**E**); p53-BPS complex (**F**).

To visually assess protein conformational changes during ligand binding, key-frame structural alignment was performed by comparing the final frame (200 ns) to the initial simulation frame (0 ns) (Figure 5). Comparison of 0 ns and 200 ns results indicates that overall protein structures remained stable without significant drift (Figure 5), supporting the validity of the observed binding poses. In the OR system, BPF retained fewer but more consistent hydrophobic contacts at 200 ns (mainly with LEU, ILE, VAL, TYR) than at 0 ns, suggesting a well-defined and persistent binding pocket (Figure 5A). BPS maintained fewer hydrophobic interactions but formed more hydrogen bonds with charged residues (ARG, GLY, LYS), suggesting a reliance on polar stabilization (Figure 5B). These findings imply that BPS depends more on dynamic polar interactions, potentially leading to weaker or less sustained binding compared to BPF [41]. In the GNB2 system, BPF maintained moderate hydrophobic contacts throughout the simulation (Figure 5C). Although BPS did not form persistent interactions with the protein by 200 ns, its stable hydrogen bond counts–protein-ligand (1.18 ± 0.04), protein-water (93.59 ± 3.63), and ligand-water (2.65 ± 0.06)–indicated a well-structured solvation shell (Figure 5D and Figure S4). This suggests that BPS may rely on dynamic solvent-mediated stabilization rather than direct binding interactions [42,43]. For p53, both ligands preserved multiple hydrophobic contacts with residues such as ILE, LYS, and LEU, and exhibited an increase in the number and extent of hydrophobic interactions throughout the simulation (Figure 5E,F). Overall, with the exception of the GNB2-BPS complex, all other systems exhibited higher binding stability during the MD simulations (Table S4).

Using the MM-PBSA method, the binding free energies (ΔG*_Binding_*) of the protein–BPF/BPS complexes were calculated (Table 2 and Table S5). The total binding free energy is composed of van der Waals interactions (ΔE*_vdw_*), electrostatic interactions (ΔE*_Ele_*), polar solvation energy (ΔG*_Polar_*), and nonpolar solvation energy (ΔG*_Nonpolar_*). The MM-PBSA results reveal that the binding free energies (ΔG*_Binding_*) of the OR-BPF system (−31.72 kcal/mol) are significantly more favorable than those of OR-BPS (−20.51 kcal/mol), suggesting stronger binding affinity for BPF. This difference is primarily driven by more favorable van der Waals interactions (ΔE*_vdw_*: −41.7 vs. −35.24 kcal/mol, BPF vs. BPS). In contrast, for GNB2 and p53 systems, the ΔG*_Binding_* values for BPF and BPS are comparable (−21.13 vs. −21.35 kcal/mol; −14.45 vs. −14.79 kcal/mol), indicating similar binding affinities. In terms of energy components, van der Waals interactions contributed the most to the total binding free energy across all complexes, with values generally exceeding −30 kcal/mol, except for the p53 system. Electrostatic interactions and nonpolar solvation energy also favored receptor-ligand binding. Overall, based on the well-converged systems (OR, GNB2, and p53), BPF demonstrated consistently stronger binding affinities than BPS. Inclusion of the additional systems (Table S5), despite their limited convergence, supports the same overall trend, suggesting that BPF generally exhibits stronger protein binding potential.

Overall, our MD simulations showed that the OR, GNB2, and p53 systems maintained stable conformations during the simulation period, forming stable receptor–ligand complexes with consistently low binding free energies (−14.45 to −31.72 kcal/mol). Energy decomposition analysis indicated that BPF generally exhibited stronger binding interactions than BPS, where this difference was mainly attributed to more favorable van der Waals contributions. This suggests that binding is primarily driven by direct dispersion interactions within relatively hydrophobic and poorly solvated pockets [44], consistent with the OR binding site’s abundance of hydrophobic residues such as LEU, ILE, VAL, and TYR. These residues likely facilitate tighter packing and more efficient dispersion interactions with BPF, enhancing complex stability and affinity [41]. In brief, BPF and BPS can form stable complexes for the three proteins, and compared with BPS, BPF has a stronger binding interaction with the proteins, indicating that it has a stronger destructive effect on the olfactory system and induces stronger toxicity [31,45]. Importantly, although the MD simulations indicate overall stability, stronger binding affinity may increase the potential risk of conformational perturbation [46,47]. Previous studies have demonstrated that small-molecule binding can lead to local or global structural rearrangements that impair protein function. For example, BPA has been shown to induce relaxation and functional changes in α-chymotrypsin [48], cause N-terminal domain rearrangement in protein-disulfide isomerase leading to impaired oxidative folding [49], and enable flunitrazepam derivatives to penetrate tight junction proteins, altering their conformation and disrupting blood–brain barrier integrity [21]. Taken together, our binding free energy analyses and MD simulations suggest that BPF exhibits stronger binding potential with key signaling pathway proteins such as OR, GNB2, and p53. This may provide mechanistic insight into BPF’s potential to cause more pronounced molecular-level disruption in the olfactory system compared to BPS.

## 4. Conclusions

In conclusion, this study demonstrates that exposure to environmentally relevant concentrations of BPF and BPS induces subtle cellular alterations in the olfactory tissues of zebrafish via molecular changes. While histopathological analyses revealed the changes in epithelial thickness, lamellar width, transcriptomic, molecular docking and molecular dynamics simulations provided computational evidence suggesting potential interactions between BPF/BPS and OR, GNB2, and p53, potentially affecting signal transduction, endocrine regulation, immune responses, and cell survival pathways. Binding free energy calculations suggest that BPF may exhibit stronger interactions than BPS. These findings highlight that even in the absence of overt tissue damage, BPF and BPS may perturb olfactory molecular pathways. Although molecular docking and molecular dynamics simulations provided computational insights into the potential interactions between BPF/BPS and olfactory-related proteins, these predictions were based on AlphaFold-generated structures and therefore require further experimental validation. Future studies should employ ligand-binding assays, receptor activity assays, or other biochemical approaches to verify these predicted protein–ligand interactions and their functional consequences. In addition, electron microscopy could be used to examine fine ultrastructural changes in the olfactory lamellae, while cell-type-specific markers could help identify p53-responsive cell populations and further investigation could assess potential sex-specific effects on olfactory function in aquatic organisms.

## Figures and Tables

**Table 1:** Measured concentrations of BPF and BPS in the exposural solutions.

Chemicals	Nominal Conc (μg/L)	Measured Conc at 0 h (μg/L)	Measured Conc After 24 h (μg/L)
Control	0	ND	ND
BPF	5.00	5.34 ± 0.30	4.90 ± 0.09
BPS	5.00	5.34 ± 0.31	5.12 ± 0.37

Note: Value are mean ± SE (*n* = 3 for each treatment). ND: not detectable.

**Table 2:** Binding free energy and energy components of receptor–ligand complexes calculated by MMPBSA.

Energy composition item (kcal/mol)	OR-BPF	GNB2-BPF	p53-BPF
ΔE*_vdw_*	−41.7	−29.56	−18.01
ΔE*_Ele_*	−4.16	−6.98	−1.99
ΔG*_Polar_*	16.69	17.73	7.06
ΔG*_Nonpolar_*	−2.55	−2.32	−1.52
ΔG*_Binding_*	−31.72	−21.13	−14.45
	OR-BPS	GNB2-BPS	p53-BPS
ΔE*_vdw_*	−35.24	−31.46	−19.21
ΔE*_Ele_*	−9.38	−8.77	−6.19
ΔG*_Polar_*	26.74	21.32	12.25
ΔG*_Nonpolar_*	−2.62	−2.44	−1.63
ΔG*_Binding_*	−20.51	−21.35	−14.79

## Data Availability

The data presented in this study are available in the article and its Supplementary Materials.

## Supplementary Materials

The following supporting information can be downloaded at: https://www.mdpi.com/article/10.3390/toxics14090776/s1, Text S1. Transcriptomic Data Processing and Quality Control; Table S1. Summary statistics of sequencing reads, base quality, and GC content; Table S2. Details of targeted proteins; Table S3. The interaction force and amino acid residue analysis of the docking between BPF/BPS and the receptor protein in *D. rerio*; Table S4. Molecular dynamics simulation of the interaction force and amino acid residue analysis of BPF/BPS docking with receptor proteins at 0ns and 200ns; Table S5. Binding free energy and energy components of protein–ligand complexes calculated by MMPBSA; Figure S1. Representative H&E-stained images of olfactory lamellae from zebrafish in different treatment groups. Three representative images were selected from each group. Columns from left to right show the Control, BPF, and BPS groups, respectively. These images were used to support the morphometric analysis of olfactory epithelial thickness and lamellar width. Scale bar as indicated; Figure S2. RMSD and radius of gyration (Rgyr) profiles of protein–ligand complexes with BPF and BPS during 200 ns molecular dynamics simulations. (A) RMSD profiles of protein backbones in BPF-bound complexes. (B) RMSD profiles of protein backbones in BPS-bound complexes. (C) Radius of gyration (Rgyr) profiles of proteins in BPF-bound complexes. (D) Radius of gyration (Rgyr) profiles of proteins in BPS-bound complexes; Figure S3. Residue-level flexibility of protein–ligand complexes with BPF and BPS during molecular dynamics simulations. RMSF profiles of proteins bound to BPF and BPS are shown for (A) CAMK2D2, (B) PDE, (C) GNG7, (D) NeuroD, (E) CREB1, (F) FOSL1, (G) CDK1, and (H) CCNB3; Figure S4. Hydrogen-bond dynamics in the GNB2–BPS complex during 200 ns molecular dynamics simulation. (A) Protein–ligand hydrogen bonds between GNB2 and BPS. (B) Protein–water hydrogen bonds between GNB2 and solvent. (C) Ligand–water hydrogen bonds between BPS and solvent.

## References

[1] Zheng Q., Xiao J., Zhang D., Li X., Xu J., Ma J., Xiao Q., Fu J., Guo Z., Zhu Y. (2024). Bisphenol analogues in infant foods in south China and implications for infant exposure. Sci. Total Environ..

[2] Cheng F., Wang J. (2024). Removal of bisphenol a from wastewater by adsorption and membrane separation: Performances and mechanisms. Chem. Eng. J..

[3] Czarny-Krzymińska K., Krawczyk B., Szczukocki D. (2023). Bisphenol A and its substitutes in the aquatic environment: Occurrence and toxicity assessment. Chemosphere.

[4] Pan Y., Xie R., Wei X., Li A.J., Zeng L. (2024). Bisphenol and analogues in indoor dust from E-waste recycling sites, neighboring residential homes, and urban residential homes: Implications for human exposure. Sci. Total Environ..

[5] Tasneem A., Akbor M.A., Rahman M.M. (2025). Assessment of bisphenol A analogues in water and sediment of an urban river system in Dhaka, Bangladesh: A regionally focused environmental investigation. RSC Adv..

[6] Yamazaki E., Yamashita N., Taniyasu S., Lam J., Lam P.K., Moon H.-B., Jeong Y., Kannan P., Achyuthan H., Munuswamy N. (2015). Bisphenol A and other bisphenol analogues including BPS and BPF in surface water samples from Japan, China, Korea and India. Ecotoxicol. Environ. Saf..

[7] Zhou N., Liu Y., Cao S., Guo R., Ma Y., Chen J. (2020). Biodegradation of bisphenol compounds in the surface water of Taihu Lake and the effect of humic acids. Sci. Total Environ..

[8] Liu J., Guo J., Cai Y., Ren J., Lu G., Li Y., Ji Y. (2022). Multimedia distribution and ecological risk of bisphenol analogues in the urban rivers and their bioaccumulation in wild fish with different dietary habits. Process Saf. Environ. Prot..

[9] Gao Y., Chen Y., Wang G.-Y., Zhan Y., Wu N.-N., Xu R., Liu S., Ji Y., Xu X.-R., Gu Y.-G. (2026). Bioaccumulation and Trophic Magnification of Endocrine-Disrupting Chemicals in an Estuarine Food Web. ACS ES T Water.

[10] Di Bella G., Litrenta F., Potortì A.G., Giacobbe S., Nava V., Puntorieri D., Albergamo A., Turco V.L. (2025). Plasticizers and Bisphenols in Sicilian Lagoon Bivalves, Water, and Sediments: Environmental Risk in Areas with Different Anthropogenic Pressure. Environments.

[11] Qiu W., Liu S., Chen H., Luo S., Xiong Y., Wang X., Xu B., Zheng C., Wang K.-J. (2021). The comparative toxicities of BPA, BPB, BPS, BPF, and BPAF on the reproductive neuroendocrine system of zebrafish embryos and its mechanisms. J. Hazard. Mater..

[12] Mu X., Liu J., Wang H., Yuan L., Wang C., Li Y., Qiu J. (2022). Bisphenol F impaired zebrafish cognitive ability through inducing neural cell heterogeneous responses. Environ. Sci. Technol..

[13] Li T., Liu B., Chen C., Liu L., Li P., Li Z.H. (2025). Insights into the effects of environmental contaminations on olfactory toxicity in fish: A latest review. Environ. Chem. Ecotoxicol..

[14] Yue N., Li D., Pan Y., Chen L., Liu S., Hou M., Luo Y. (2025). Structure, transduction pathway, behavior and toxicity of fish olfactory in aquatic environments. Comp. Biochem. Physiol. Part C Toxicol. Pharmacol..

[15] Zeng S., Tong C., Yang F. (2025). Hexafluoropropylene oxide trimer acid induces olfactory toxicity to crucian carp by disrupting olfactory function and olfactory-mediated behavior. Aquat. Toxicol..

[16] Takesono A., Dimitriadou S., Clark N.J., Handy R.D., Mourabit S., Winter M.J., Kudoh T., Tyler C.R. (2023). Zinc oxide nanoparticles disrupt development and function of the olfactory sensory system impairing olfaction-mediated behaviour in zebrafish. Environ. Int..

[17] Al-Zahaby S.A., Farag M.R., Alagawany M., Taha H.S., Varoni M.V., Crescenzo G., Mawed S.A. (2023). Zinc oxide nanoparticles (ZnO-NPs) induce cytotoxicity in the zebrafish olfactory organs via activating oxidative stress and apoptosis at the ultrastructure and genetic levels. Animals.

[18] Huang L., Zhang W., Tong D., Lu L., Zhou W., Tian D., Liu G., Shi W. (2023). Triclosan and triclocarban weaken the olfactory capacity of goldfish by constraining odorant recognition, disrupting olfactory signal transduction, and disturbing olfactory information processing. Water Res..

[19] Takesono A., Schirrmacher P., Scott A., Green J.M., Lee O., Winter M.J., Kudoh T., Tyler C.R. (2022). Estrogens regulate early embryonic development of the olfactory sensory system via estrogen-responsive glia. Development.

[20] Wu J., Lv J., Zhao L., Zhao R., Gao T., Xu Q., Liu D., Yu Q., Ma F. (2023). Exploring the role of microbial proteins in controlling environmental pollutants based on molecular simulation. Sci. Total Environ..

[21] Lin W., Qin Y., Ren Y. (2024). Flunitrazepam and its metabolites induced brain toxicity: Insights from molecular dynamics simulation and transcriptomic analysis. J. Hazard. Mater..

[22] Li D., Xie L., Carvan M.J., Guo L. (2019). Mitigative effects of natural and model dissolved organic matter with different functionalities on the toxicity of methylmercury in embryonic zebrafish. Environ. Pollut..

[23] Klopfenstein D.V., Zhang L., Pedersen B.S., Ramírez F., Vesztrocy A.W., Naldi A., Mungall C.J., Yunes J.M., Botvinnik O., Weigel M. (2018). GOATOOLS: A Python library for Gene Ontology analyses. Sci. Rep..

[24] Xie C., Mao X., Huang J., Ding Y., Wu J., Dong S., Kong L., Gao G., Li C.-Y., Wei L. (2011). KOBAS 2.0: A web server for annotation and identification of enriched pathways and diseases. Nucleic Acids Res..

[25] Adasme M.F., Linnemann K.L., Bolz S.N., Kaiser F., Salentin S., Haupt V.J., Schroeder M. (2021). PLIP 2021: Expanding the scope of the protein–ligand interaction profiler to DNA and RNA. Nucleic Acids Res..

[26] Huang L., Zhang W., Han Y., Tang Y., Zhou W., Liu G., Shi W. (2022). Anti-depressant fluoxetine hampers olfaction of goldfish by interfering with the initiation, transmission, and processing of olfactory signals. Environ. Sci. Technol..

[27] Lu L., Shan C., Tong D., Yu Y., Zhang W., Zhang X., Shu Y., Li W., Liu G., Shi W. (2024). Olfactory toxicity of tetrabromobisphenol A to the goldfish Carassius auratus. J. Hazard. Mater..

[28] Razmara P., Pyle G.G. (2021). Effect of copper nanoparticles and copper ions on the architecture of rainbow trout olfactory mucosa. Ecotoxicol. Environ. Saf..

[29] Singh R., Pandey P.N. (2015). Molecular docking and molecular dynamics study on SmHDAC1 to identify potential lead compounds against Schistosomiasis. Mol. Biol. Rep..

[30] Enyoh C.E., Wang Q., Ovuoraye P.E., Maduka T.O. (2022). Toxicity evaluation of microplastics to aquatic organisms through molecular simulations and fractional factorial designs. Chemosphere.

[31] Pei J., Peng J., Wu M., Zhan X., Wang D., Zhu G., Wang W., An N., Pan X. (2025). Analyzing the potential targets and mechanisms of chronic kidney disease induced by common synthetic Endocrine Disrupting Compounds (EDCs) in Chinese surface water environment using network toxicology and molecular docking techniques. Sci. Total Environ..

[32] Yao H., Ke H., Zhang X., Pan S.-J., Li M.-S., Yang L.-P., Schreckenbach G., Jiang W. (2018). Molecular recognition of hydrophilic molecules in water by combining the hydrophobic effect with hydrogen bonding. J. Am. Chem. Soc..

[33] Khemis I.B., Aouaini F., Bukhari L., Alruwaili A., Knani S., Lamine A.B. (2024). Quantitative investigations of Zebrafish olfactory receptor ORA1 responsiveness to three pheromones: Microscopic and macroscopic characterizations via an advanced statistical physics treatment. Int. J. Biol. Macromol..

[34] Pace C.N., Fu H., Fryar K.L., Landua J., Trevino S.R., Shirley B.A., Hendricks M.M., Iimura S., Gajiwala K., Scholtz J.M. (2011). Contribution of hydrophobic interactions to protein stability. J. Mol. Biol..

[35] Mahadevi A.S., Sastry G.N. (2013). Cation−π interaction: Its role and relevance in chemistry, biology, and material science. Chem. Rev..

[36] Lin W., Yan Y., Ping S., Li P., Li D., Hu J., Liu W., Wen X., Ren Y. (2020). Metformin-induced epigenetic toxicity in zebrafish: Experimental and molecular dynamics simulation studies. Environ. Sci. Technol..

[37] Hawkins P.C. (2017). Conformation generation: The state of the art. J. Chem. Inf. Model..

[38] Khairul Anuar N.F.S., Wahab R.A., Huyop F., Normi Y.M., Oyewusi H.A., Susanti E. (2024). In silico mutagenesis on active site residues of Acinetobacter haemolyticus lipase KV1 for improved binding to polyethylene terephthalate (PET). J. Biomol. Struct. Dyn..

[39] Liu K., Watanabe E., Kokubo H. (2017). Exploring the stability of ligand binding modes to proteins by molecular dynamics simulations. J. Comput.-Aided Mol. Des..

[40] Wu S.-J., Feng R., Meng R., Ji Q.-Y., Tao H., Xu B.-C., Zhang B. (2025). Exploring the binding mechanism and functional properties of lactoferrin-berberine complex: Based on multispectral analysis, molecular docking, and dynamics simulations. Food Chem..

[41] Desantis F., Miotto M., Di Rienzo L., Milanetti E., Ruocco G. (2022). Spatial organization of hydrophobic and charged residues affects protein thermal stability and binding affinity. Sci. Rep..

[42] Reichmann D., Phillip Y., Carmi A., Schreiber G. (2008). On the contribution of water-mediated interactions to protein-complex stability. Biochemistry.

[43] Bellissent-Funel M.-C., Hassanali A., Havenith M., Henchman R., Pohl P., Sterpone F., Van Der Spoel D., Xu Y., Garcia A.E. (2016). Water determines the structure and dynamics of proteins. Chem. Rev..

[44] Barratt E., Bingham R.J., Warner D.J., Laughton C.A., Phillips S.E., Homans S.W. (2005). Van der Waals interactions dominate ligand− protein association in a protein binding site occluded from solvent water. J. Am. Chem. Soc..

[45] Trott O., Olson A.J. (2010). AutoDock Vina: Improving the speed and accuracy of docking with a new scoring function, efficient optimization, and multithreading. J. Comput. Chem..

[46] Seo M.-H., Park J., Kim E., Hohng S., Kim H.-S. (2014). Protein conformational dynamics dictate the binding affinity for a ligand. Nat. Commun..

[47] Vögele M., Zhang B.W., Kaindl J., Wang L. (2023). Is the functional response of a receptor determined by the thermodynamics of ligand binding?. J. Chem. Theory Comput..

[48] Guo S., Zhao Q., Li Y., Chu S., He F., Li X., Sun N., Zong W., Liu R. (2023). Potential toxicity of bisphenol A to α-chymotrypsin and the corresponding mechanisms of their binding. Spectrochim. Acta Part A Mol. Biomol. Spectrosc..

[49] Okumura M., Kadokura H., Hashimoto S., Yutani K., Kanemura S., Hikima T., Hidaka Y., Ito L., Shiba K., Masui S. (2014). Inhibition of the functional interplay between endoplasmic reticulum (ER) oxidoreduclin-1α (Ero1α) and protein-disulfide isomerase (PDI) by the endocrine disruptor bisphenol A. J. Biol. Chem..

