# Supplementary material for: Unveiling the Molecular Mechanisms of Bisphenol-Induced Olfactory Toxicity in Zebrafish
Source: Toxics. 2026 Aug 31;14(9):776. doi: 10.3390/toxics14090776 (PMC13611145; doi:10.3390/toxics14090776)

Supplementary materials

Unveiling the Molecular Mechanisms of Bisphenol-Induced Olfactory Toxicity in Zebrafish

Yaxuan Li [1,2], Jing Cao [1,2], Haoliang Jiang [1,2], Mei Hou [1,2], Xuefeng Hu [1,2], Liting Chen [3], Dan Li [1,2,*] and Yongju Luo [3]

1 The Research Institution of Beautiful China and Ecological Civilization (A University Think Tank of Shanghai Municipality), Shanghai Institute of Technology, Shanghai 201418, China; liyaxuan127@163.com (Y.L.); 15629343669@163.com (J.C.); jhl2622744588@163.com (H.J.); hm13275326626@163.com (M.H.); xuefenghu@163.com (X.H.)
2 Guangxi Academy of Fishery Sciences, Nanning 530021, China
3 Faculty of Urban Construction and Ecological Technology, Shanghai Institute of Technology, Shanghai 201418, China; chenlt3433@126.com (L.C.); lfylzc123@163.com (Y.L.)
* Correspondence: ldanltm@163.com

Academic Editor: Lisa Truong

Received: 18 July 2026
Revised: 14 August 2026
Accepted: 26 August 2026
Published: 31 August 2026

Copyright: © 2026 by the authors. Submitted for possible open access publication under the terms and conditions of the Creative Commons Attribution (CC BY) license.

Text S1 Transcriptomic Data Processing and Quality Control

The raw data were filtered, and low-quality, ambiguous and adapter sequences were removed by fastp to obtain clean reads. Then, the Q20, Q30, and GC content of the clean data were calculated.Then clean reads were separately aligned to reference genome (http://asia.ensembl.org/Danio_rerio/Info/Index) with orientation mode using HISAT2 software (http:// ccb. jhu. edu/ softw are/ hisat2/ index. shtml). Ten mapped reads of each sample were assembled by StringTie (https:// ccb. jhu. edu/ softw are/ strin gtie/ index. shtml?t= examp le) in a reference-based approach. RSEM (http:// dewey lab. biost at. wisc. edu/ rsem/) was used to quantify gene abundances. Differential expression analysis was performed using DESeq2. The threshold for signiffcantly differentially expressed genes (DEGs) was set at a |log2(Fold change)| ⩾ 1 and p-value ⩽0.05. Gene Ontology (GO) function and Kyoto Encyclopedia of Genes and Genomes (KEGG) pathway enrichment were analyzed using the Majorbio cloud platform (https://www.majorbio.com).

Table S1 Summary statistics of sequencing reads, base quality, and GC content

Sample	Raw reads	Raw bases	Clean reads	Clean bases	Error rate(%)	Q20(%)	Q30(%)	GC content(%)
C_1	44307300	6690402300	42513698	6360070040	0.0246	98.08	94.65	44.29
C_2	43989004	6642339604	42511398	6368064973	0.0237	98.48	95.54	46.49
C_3	44941458	6786160158	42839912	6384227747	0.0246	98.06	94.81	41.08
F_1	49378660	7456177660	43705890	6444365180	0.0250	97.88	94.42	44.93
F_2	46379242	7003265542	42899738	6344916718	0.0242	98.29	95.06	44.60
F_3	44599748	6734561948	43276834	6421134444	0.0245	98.2	94.78	44.90
S_1	46506604	7022497204	45421428	6782174487	0.0242	98.32	94.96	42.15
S_2	49311104	7445976704	41418318	6117896691	0.0259	97.59	93.40	44.52
S_3	49201134	7429371234	45433446	6751659589	0.0236	98.53	95.73	45.45

Table S2 Details of targeted proteins

Pathway	Name	Abbr.	UniProt ID	Average pLDDT
Signal transduction	Phosphodiesterase	PDE	A0A2R8RXK7	78.81
	Calcium/calmodulin-dependent protein kinase II delta 2	CAMK2D2	Q6DGS3	84.19
	Neurogenic differentiation factor	NeuroD	F1QWM8	63.47
	G protein subunit beta 2	GNB2	Q5BJ11	96.38
	Guanine nucleotide-binding protein subunit gamma 7	GNG7	Q6DGZ5	95.59
	Olfactory receptor	OR	Q2PRG5	87.94
Cell growth and death pathway	Cyclin-dependent kinase 1	CDK1	M9TJA4	68
	G2/mitotic-specific cyclin-B3	CCNB3	Q08C91	79.12
	Cellular tumor antigen p53	p53	P79734	76.75
Immune system pathway	FOS-like 1, AP-1 transcription factor subunit beta	FOSL1	E9QHS1	67.62
Endocrine system pathway	Cyclic AMP-responsive element-binding protein 1b	CREB1	Q803K8	61.38

Table S3 The interaction force and amino acid residue analysis of the docking between BPF/ BPS and the receptor protein in *D. rerio*

Ligand	Protein	Binding energy (kcal/mol)	Bonding (number)	Interaction site (distance, Å)
BPF	OR	-8.3	Hydrophobic Interactions (8)	TYR107 (3.32)<br>LEU111 (3.41)<br>LEU161 (3.76)<br>ILE203 (3.55)<br>ILE204 (3.50)<br>TYR207 (3.64)<br>LEU253 (3.54)<br>VAL257 (3.39)
			Hydrogen Bonds (2)	ASN182 (3.17)<br>ARG261 (2.19)
	GNB2	-6.3	Hydrogen Bonds (4)	SER147 (2.29)<br>ALA232 (2.99)<br>ALA232 (2.21)<br>VAL276 (3.34)
	p53	-5.9	Hydrophobic Interactions (6)	GLU15 (3.72)<br>ARG16 (3.56)<br>LYS22 (3.76)<br>LYS22 (3.82)<br>LYS22 (3.65)<br>LEU27 (3.57)
			Hydrogen Bonds (2)	ARG16 (2.02)<br>ASP31 (2.87)
	CAMK2D2	-7.1	Hydrophobic Interactions (7)	LEU479 (3.59)<br>VAL486 (3.77)<br>TYR513 (3.53)<br>TYR513 (3.52)<br>TYR513 (3.97)<br>VAL551 (3.76)<br>PRO552 (3.81)
			Hydrogen Bonds (1)	ASN554 (2.26)
	PDE	-6.9	Hydrophobic Interactions (6)	ILE351 (3.61)<br>LEU368 (3.58)<br>LEU368 (3.57)<br>PHE372 (3.83)<br>LEU389 (3.63)<br>PHE404 (3.54)
			Hydrogen Bonds (4)	TYR202 (2.45)<br>TYR202 (2.58)<br>SER400 (2.900<br>GLN401 (2.57)
			π-Stacking (1)	PHE404 (4.06)
	GNG7	-4.6	Hydrophobic Interactions (2)	LEU35 (3.69)<br>TYR38 (3.64)
			Hydrogen Bonds (1)	GLN41 (2.41)
			π-Stacking (2)	TYR38 (3.73)<br>TYR38 (4.87)
	NeuroD	-5.3	Hydrophobic Interactions (2)	ALA181 (3.68)<br>LEU186 (3.84)
	CREB1	-5.4	Hydrophobic Interactions (1)	TYR221 (3.78)
			π-Stacking (2)	TYR221 (4.82)<br>TYR221 (3.79)

	FOSL1	-5.3	Hydrophobic Interactions (5)	ILE52 (3.51)<br>ILE52 (3.61)<br>LEU58 (3.77)<br>PHE61 (3.61)<br>LEU62 (3.94)
			Hydrogen Bonds (1)	LEU49 (3.24)
			π-Stacking (1)	PHE61 (3.72)
	CDK1	-6.5	Hydrophobic Interactions (4)	PHE44 (3.68)<br>GLN45 (3.85)<br>PHE48 (3.64)<br>PHE118 (3.70)
			π-Stacking (3)	PHE44 (3.90)<br>PHE48 (4.07)<br>PHE118 (4.22)
	CCNB3	-5.7	Hydrophobic Interactions (6)	GLU183 (3.67)<br>GLU183 (3.86)<br>GLU186 (3.41)<br>GLU186 (3.80)<br>VAL369 (3.14)<br>LYS373 (3.72)
			Hydrogen Bonds (2)	ASN187 (2.58)<br>ASN187 (2.27)
BPS	OR	-7.1	Hydrophobic Interactions (3)	GLU176 (3.90)<br>LYS187 (3.61)<br>TYR259 (3.92)
			Hydrogen Bonds (2)	ALA174 (2.21)<br>GLY263 (2.33)
			π-Cation Interactions (1)	ARG272 (4.28)
	GNB2	-6.9	Hydrophobic Interactions (1)	ALA231 (3.85)
			Hydrogen Bonds (2)	ALA232 (3.00)<br>VAL276 (2.43)
	p53	-5.9	Hydrophobic Interactions (3)	GLU15 (3.56)<br>LEU27 (3.68)<br>ASP31 (3.92)
			Hydrogen Bonds (1)	ARG16 (2.15)
	CAMK2D2	-7.3	Hydrophobic Interactions (7)	LEU479 (3.54)<br>VAL486 (3.57)<br>THR511 (3.67)<br>TYR513 (3.57)<br>TYR513 (3.48)<br>VAL551 (3.71)<br>PRO552 (3.77)
			Hydrogen Bonds (1)	SER550 (2.28)
	PDE	-6.2	Hydrophobic Interactions (3)	TYR130 (3.65)<br>ILE135 (3.66)<br>LYS139 (3.55)
	GNG7	-4.7	Hydrophobic Interactions (4)	GLN9 (3.69)<br>LYS12 (3.57)<br>LEU13 (3.79)<br>LEU13 (3.55)
			Hydrogen Bonds (1)	LYS12 (2.89)
	NeuroD	-5.7	Hydrophobic Interactions (2)	TRP21 (3.65)<br>LEU113 (3.69)
			Hydrogen Bonds (1)	ARG109 (2.27)
			π-Stacking (2)	TRP21 (3.67)<br>TRP21 (3.96)
	CREB1	-5.4	Hydrophobic Interactions (1)	TYR221 (3.71)
			Hydrogen Bonds (2)	ALA219 (2.23)<br>GLN224 (2.25)

		π-Stacking (2)	TYR221 (4.99)<br>TYR221 (3.88)
FOSL1	-5.1	Hydrophobic Interactions (5)	ILE52 (3.48)<br>ILE52 (3.71)<br>ILE52 (3.95)<br>LEU58 (3.90)<br>PHE61 (3.77)
		Hydrogen Bonds (1)	LEU49 (2.17)
		π-Stacking (1)	PHE61 (3.77)
CDK1	-6.4	Hydrophobic Interactions (4)	PHE44 (3.71)<br>GLN45 (3.71)<br>PHE48 (3.53)<br>PHE118 (3.79)
		π-Stacking (3)	PHE44 (3.79)<br>PHE48 (3.97)<br>PHE118 (4.31)
CCNB3	-6.6	Hydrophobic Interactions (4)	ALA282 (3.61)<br>LYS283 (3.66)<br>LYS283 (3.62)<br>LYS283 (3.69)
		Hydrogen Bonds (2)	ARG280 (2.14)<br>LYS283 (3.11)

Table S4 Molecular dynamics simulation of the interaction force and amino acid residue analysis of BPF/BPS docking with receptor proteins at 0ns and 200ns

Ligand	Protein	Bonding (number)	Interaction site (distance, Å)
BPF (0 ns)	OR	Hydrophobic Interactions (8)	TYR107 (3.90)<br>ILE203 (3.76)<br>ILE204 (3.67)<br>ILE204 (3.57)<br>TYR207 (3.60)<br>LEU253 (3.89)<br>LEU254 (3.86)<br>VAL257 (3.23)
		Hydrogen Bonds (1)	ARG261 (3.45)
	GNB2	Hydrophobic Interactions (2)	ASN230 (3.69)<br>ALA231 (3.81)
	p53	Hydrophobic Interactions (5)	GLU15 (3.58)<br>ILE19 (3.58)<br>LYS22 (3.51)<br>ASP25 (3.81)<br>LEU27 (3.88)
BPS (0 ns)	OR	Hydrophobic Interactions (4)	GLU176 (3.58)<br>LYS177 (3.79)<br>LYS187 (3.57)<br>TYR259 (3.38)
		π-Cation Interactions (1)	ARG272 (4.36)
	GNB2	Hydrophobic Interactions (2)	ASN230 (3.83)<br>ALA231 (3.30)
		Hydrogen Bonds (2)	ALA232 (2.73)<br>VAL276 (2.79)
	p53	Hydrophobic Interactions (3)	ILE19 (3.89)<br>LYS22 (3.86)<br>LEU27 (3.68)
		π-Cation Interactions (1)	LYS22 (5.69)
BPF (200 ns)	OR	Hydrophobic Interactions (6)	ILE203 (3.49)<br>ILE204 (3.47)<br>TYR207 (3.49)<br>LEU253 (3.72)<br>LEU253 (3.92)<br>VAL257 (3.75)
		Hydrogen Bonds (1)	TYR207 (2.68)
	GNB2	Hydrophobic Interactions (2)	MET188 (3.63)<br>ASN230 (3.93)
	p53	Hydrophobic Interactions (6)	GLU18 (3.87)<br>ILE19 (3.83)<br>ILE19 (3.84)<br>LYS22 (3.48)<br>LYS22 (3.37)<br>LEU23 (3.60)
BPS (200 ns)	OR	Hydrophobic Interactions (2)	ARG272 (3.52)<br>TYR259 (3.64)
		Hydrogen Bonds (4)	GLY18 (3.15)<br>GLY263 (2.90)<br>LYS187 (3.06)<br>TYR262 (2.93)
	GNB2	-	-
	p53	Hydrophobic Interactions (6)	GLU15 (3.72)<br>ARG16 (3.65)<br>ILE19 (3.88)<br>LYS22 (3.63)<br>ASP25 (3.79)<br>LEU29 (3.82)

Table S5 Binding free energy and energy components of protein–ligand complexes calculated by MMPBSA

Energy composition item （kcal/mol）	CAMK2D2-BPS	PDE-BPS	GNG7-BPS	NeuroD-BPS	CREB1-BPS	FOSL1-BPS	CDK1-BPS	CCNB3-BPS
ΔE_{vdw}	-45.15	-36.93	-40.3	-32.13	-45.46	–	-44.68	-34.13
ΔE_{Ele}	-7.34	-9.87	-8.91	-10.19	-11.2	–	-10.92	-6.74
ΔG_{Polar}	24.05	28.9	21.64	22.72	22.29	–	23.35	17.93
$\Delta G_{Nonpolar}$	-2.76	-2.69	-2.59	-2.47	-2.68	–	-2.72	-2.53
$\Delta G_{Binding}$	-31.2	-20.59	-30.16	-22.08	-37.04	–	-34.97	-25.47
	CAMK2D2-BPF	PDE-BPF	GNG7-BPF	NeuroD-BPF	CREB1-BPF	FOSL1-BPF	CDK1-BPF	CCNB3-BPF
ΔE_{vdw}	-40.03	-36.44	-32.99	-42.96	-42.37	-42.39	-33.81	-36.11
ΔE_{Ele}	-8.93	-4.94	-4.61	-9.85	-6.03	-4.87	-5.2	-8.9
ΔG_{Polar}	25.12	13.36	12.65	20.55	19.45	18.86	13.76	23.3
$\Delta G_{Nonpolar}$	-2.54	-2.33	-2.21	-2.45	-2.46	-2.38	-2.3	-2.49
$\Delta G_{Binding}$	-26.39	-30.35	-27.16	-34.7	-31.4	-30.79	-27.55	-24.2

Figure S1 Representative H&E-stained images of olfactory lamellae from zebrafish in different treatment groups. Three representative images were selected from each group. Columns from left to right show the Control, BPF, and BPS groups, respectively. These images were used to support the morphometric analysis of olfactory epithelial thickness and lamellar width. Scale bar as indicated.

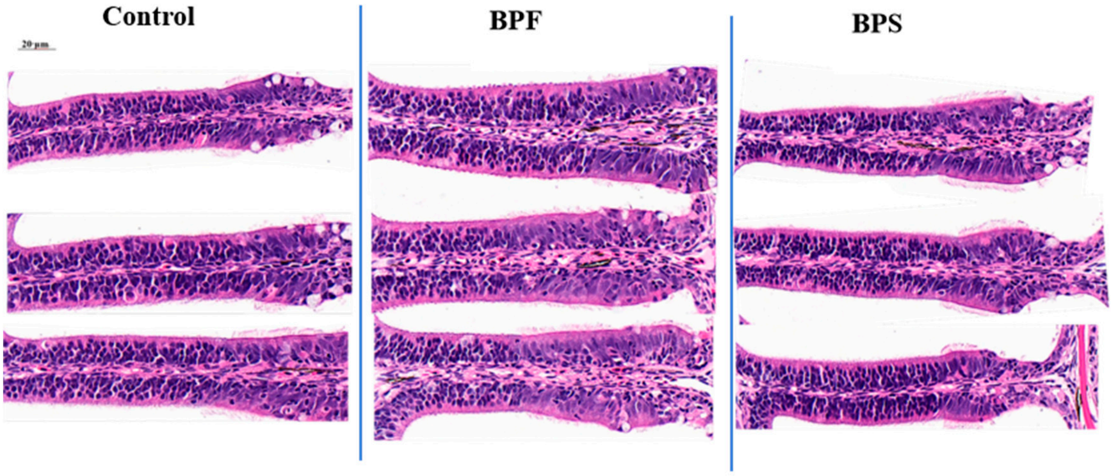

Figure S2. RMSD and radius of gyration (Rgyr) profiles of protein–ligand complexes with BPF and BPS during 200 ns molecular dynamics simulations. (A) RMSD profiles of protein backbones in BPF-bound complexes. (B) RMSD profiles of protein backbones in BPS-bound complexes. (C) Radius of gyration (Rgyr) profiles of proteins in BPF-bound complexes. (D) Radius of gyration (Rgyr) profiles of proteins in BPS-bound complexes.

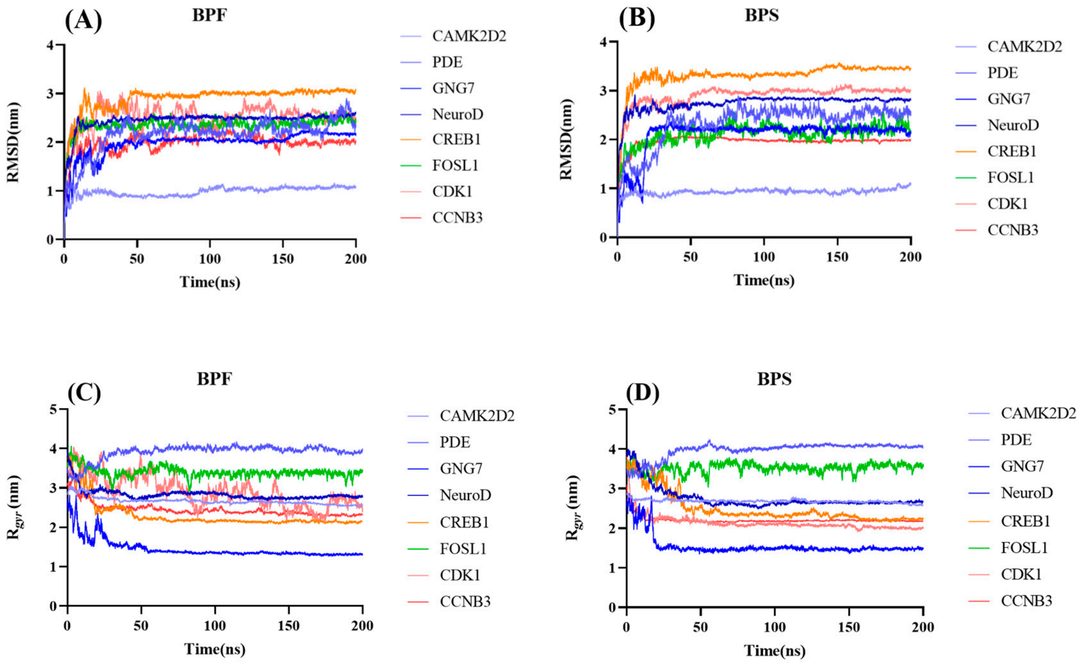

Figure S3. Residue-level flexibility of protein–ligand complexes with BPF and BPS during molecular dynamics simulations. RMSF profiles of proteins bound to BPF and BPS are shown for (A) CAMK2D2, (B) PDE, (C) GNG7, (D) NeuroD, (E) CREB1, (F) FOSL1, (G) CDK1, and (H) CCNB3.

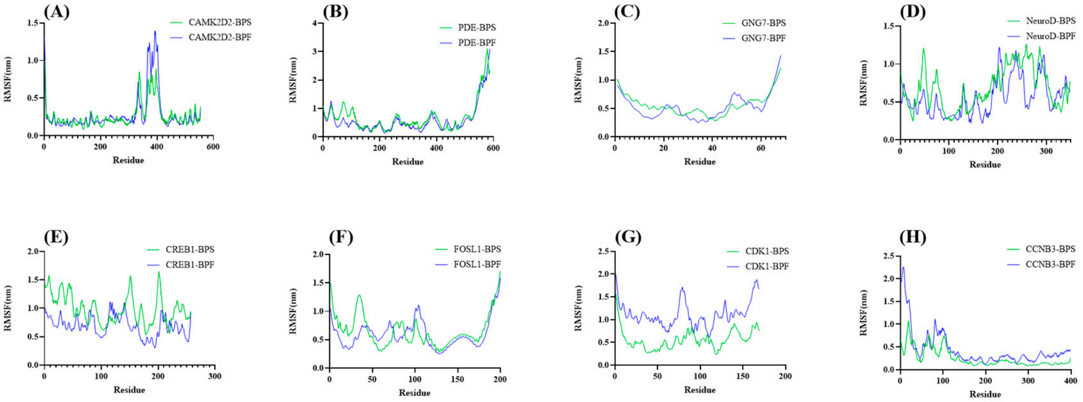

Figure S4. Hydrogen-bond dynamics in the GNB2–BPS complex during 200 ns molecular dynamics simulation. (A) Protein–ligand hydrogen bonds between GNB2 and BPS. (B) Protein–water hydrogen bonds between GNB2 and solvent. (C) Ligand–water hydrogen bonds between BPS and solvent.

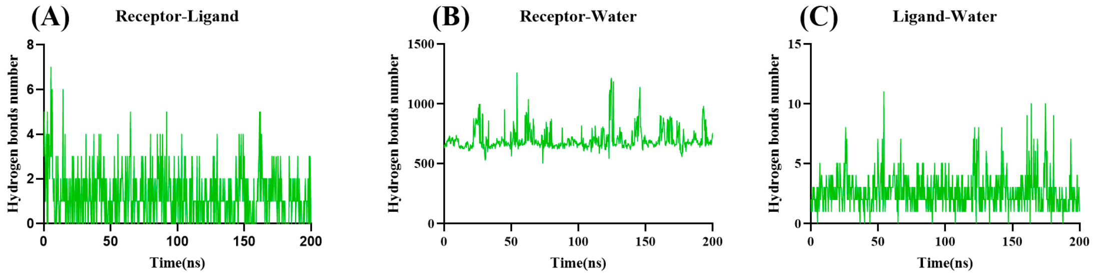

Supplement: Supplementary file 1 [file toxics-14-00776-s001.zip › toxics-4477524-supplementary.pdf]
